# ASO Author Reflections: Neoadjuvant Treatment Does not Increase Postoperative Complications Compared with Upfront Surgery in Esophageal Cancer

**DOI:** 10.1245/s10434-025-18220-w

**Published:** 2025-09-05

**Authors:** Ville E. J. Sirviö, Jari V. Räsänen, Olli Helminen, Mika Helmiö, Heikki Huhta, Raija Kallio, Vesa Koivukangas, Arto Kokkola, Elina Lietzen, Sanna Meriläinen, Vesa-Matti Pohjanen, Tuomo Rantanen, Ari Ristimäki, Juha Saarnio, Eero Sihvo, Tuula Tyrväinen, Mikko Uimonen, Antti Valtola, Joonas H. Kauppila

**Affiliations:** 1 Department of Oesophageal and General Thoracic Surgery, Helsinki University Hospital and University of Helsinki, Helsinki, Finland; 2 Surgery Research Unit, Oulu University Hospital and University of Oulu, Oulu, Finland; 3 The Division of Digestive Surgery and Urology, Turku University Hospital and University of Turku, Turku, Finland; 4 Department of Oncology and Radiotherapy, Oulu University Hospital, Oulu, Finland; 5 Department of Surgery, University of Helsinki and Helsinki University Hospital, Helsinki, Finland; 6 Cancer and Translational Medicine Research Unit, Medical Research Center Oulu, University of Oulu and Oulu University Hospital, Oulu, Finland; 7 Department of Surgery, University of Eastern Finland and Kuopio University Hospital, Kuopio, Finland; 8 Department of Pathology and HUS Diagnostic Center, University of Helsinki and Helsinki University Hospital, Helsinki, Finland; 9 Applied Tumor Genomics Research Program, Research Programs Unit, University of Helsinki, Helsinki, Finland; 10 Department of Surgery, Central Finland Central Hospital, Jyvaskyla, Finland; 11 Department of Gastroenterology and Alimentary Tract Surgery, Tampere University Hospital, Tampere, Finland; 12 Heart Hospital, Tampere University Hospital, Wellbeing Services County of Pirkanmaa, Tampere, Finland; 13 Upper Gastrointestinal Surgery, Department of Molecular Medicine and Surgery, Karolinska Institutet, Stockholm, Sweden

## Past

The role of neoadjuvant treatment (nT) and its modalities (chemotherapy [nCT], chemoradiotherapy [nCRT]) in the risk of complications after esophagectomy for cancer is unclear outside clinical trial settings. Randomized studies to date have not reported that nT increases surgical complications, however meta-analyses and the only existing nationwide study have had opposing results.^1,2^ The latest randomized data comparing nCRT and nCT in surgical risk have also had mixed results.^3,4^ Further large, population-based studies are warranted.

## Present

We compared postoperative complications and 90-day mortality after nT and upfront surgery, and after chemoradiotherapy and chemotherapy, in an unselected, population-based, nationwide cohort using standardized outcome definitions.^5^ There were no meaningful differences in postoperative outcomes in patients undergoing nT versus upfront surgery or patients undergoing nCRT versus nCT. Outcomes were also similar in patients with aborted or dose-reduced neoadjuvant regimens versus patients tolerating full intended neoadjuvant regimens.

## Future

In the present study, the choice of preoperative treatment strategy did not affect the risk of postoperative complications or mortality. These results support the use of preoperative oncologic treatment, with the choice of modality guided primarily by oncologic and survival outcomes, without concerns of said choice affecting surgical risk. Patients experiencing adverse events during nT are often considered to be of higher surgical risk and can be denied surgery. Similarly, nT may be withheld from patients with reduced performance status due to fear of complications. Our results suggest these concerns are unwarranted and such patients should not be excluded from surgery or nT if otherwise eligible.

Further population-based studies using widely accepted, standardized outcome definitions are needed for international comparison, identification of potential pitfalls, and to facilitate quality-improvement efforts. Future studies should monitor more recently introduced neoadjuvant regimens such as fluorouracil, leucovorin, oxaliplatin and docetaxel (FLOT), and emerging neoadjuvant immunotherapies in surgical safety.
